# Perceived Discrimination, Psychological Distress and Cardiovascular Risk in Migrants in Spain

**DOI:** 10.3390/ijerph17124601

**Published:** 2020-06-26

**Authors:** María José Martos-Méndez, Alba García-Cid, Luis Gómez-Jacinto, Isabel Hombrados-Mendieta

**Affiliations:** 1 Faculty of Psychology, University of Málaga, 29071 Málaga, Spain; garciacidalba@uma.es (A.G.-C.); mihombrados@uma.es (I.H.-M.); 2 Faculty of Social and Labour Studies, University of Málaga, 29071 Málaga, Spain; jacinto@uma.es

**Keywords:** cardiovascular risk factors, immigrants, discrimination, psychological distress, cardiovascular disease

## Abstract

The aim of the present study is to determine the effect of discrimination and psychological distress on the cardiovascular health of immigrants, as well as to analyse potential differences based on age, gender, length of residence in host country and geographic origin. The sample was formed by 1714 immigrants from Africa, Eastern Europe and Latin America. Of the sample, 48.7% were men and 51.3% were women. Most relevant results show that discrimination (*t* = 4.27; *p* = 0.000) and psychological distress (*t* = 4.35; *p* = 0.000) experienced by immigrants predict their cardiovascular risk. Furthermore, psychological distress mediates the relation between discrimination and risk (*t* = 4.03; *p* = 0.000). Significant differences between men and women were found, as well as differences based on ethnicity, although to a lesser extent. Age affects the relation between discrimination, psychological distress and arterial hypertension and hypercholesterolemia. Results are notably relevant for the design of preventive health programmes for immigrants and intervention strategies in order to prevent diseases that may imply cardiovascular risks and seriously affect immigrants’ health.

## 1. Introduction

### 1.1. Perceived Discrimination and Cardiovascular Risk

Hypertension and hyperlipidemia are common diseases worldwide [1], and they are considered two of the main risk factors of cardiovascular problems [2]. Complications caused by these diseases, such as myocardial infarction and cerebrovascular accident, are the main causes of death and disability among immigrants and native populations [3].

Upon the arrival of immigrants to host countries, they usually experience different sorts of discrimination that make them feel unjustly treated just because of having a different ethnical origin [4]. These feelings of exclusion and rejection are a strong source of stress that affects their health severely. The perception of discrimination is tightly linked to the risk of suffering cardiovascular diseases [5], even when sociodemographic variables such as socioeconomic levels are taken into consideration [6]. In fact, ethnical discrimination is said to be linked to cardio-metabolic diseases, high blood pressure and obesity, among others [7,8].

With reference to the discrimination of immigrants, Ikram et al. (2015) conveyed a study on the mortality rate of immigrant collectives based on the different integration policies in different countries [9]. According to these authors, there is a clear relation between high mortality rates and cardiovascular diseases in countries with “excluding” models such as Denmark. It has also been suggested that the density of migrant communities in host countries is related to cardiovascular-risk diseases. Denser communities would act as protective factors of mortality against discrimination in minoritarian collectives such as US Blacks and Hispanics [10]. This means that further analysis on how the lack of integration in communities affects immigrants and, therefore, the impact of discrimination on cardiovascular-risk diseases should be further analysed.

### 1.2. Health, Psychological Distress and Cardiovascular Risk

Immigrants often find it more difficult to access health services, probably because they are a group in risk of social exclusion and economically disadvantaged [11,12]. Therefore, they have less access to preventive and early detection test [13]. Language and cultural barriers contribute to make this situation more difficult for immigrants [14,15]. Consequently, immigrants are more likely to be in higher risk due to not having been diagnosed or because conditions such as hypertension and dyslipidemia are not being followed [16]. Likewise, those individuals who have limited social networks and low levels of integration within communities may be the ones who struggle more to handle health issues or obtain health-related information from their support networks. This situation often occurs with immigrants, who experience strong cultural differences [17,18].

The challenges faced by immigrants in their new psychosocial and cohabitation contexts are highly stressful experiences that imply, as confirmed by several studies, a higher risk of suffering stress and, frequently, mental disorders [19,20]. Research conveyed by Rosenthal (2014) concluded that the migration process has negative effects on the later appearance of hypertension and other cardiovascular-risk factors in immigrants [21]. Therefore, only the stress caused by the acculturative process leads to higher cardiovascular risk [22]. Psychological distress is a risk factor that may well be mediating the effect of discrimination experienced by immigrants and the risk of suffering hypertension and dyslipidemia, both considered cardiovascular-risk factors. Psychological distress would thus promote the beginning, progression and continuation of some cardiovascular diseases such as atrial fibrillation through mechanisms that rocket the levels of the autonomic nervous system and alter the articular substrate, caused by chronic stress and anxiety states [23]. Besides, as immigrants settle in the host country and adopt new behaviours such as smoking, drinking alcohol or eating fast food, the risk of developing cardiovascular diseases, diabetes and obesity increases [24]. For instance, western nutrition habits, such as deficient Magnesium (Mg2+), are related to health problems and cardiovascular and metabolic risks [25]. This is because Magnesium (Mg2+) is linked to good blood circulation, it maintains the vascular and metabolic homeostasis, and it regulates the processes of oxidation and inflammation responsible for triggering cardiovascular diseases and atherogenesis [26]. Therefore, alongside chronic stress and psychological factors, poor nutrition habits are also a main risk factor that affects the normal functioning of the immune system, and it produces inflammatory reactions [27,28].

### 1.3. Sociodemographic Variables and Cardiovascular Risk

The revised studies on immigrant populations show that the longer immigrants live in host countries, the more their health decreases and matches the rate of diseases of the native population. Nevertheless, other studies also suggest that the decline in immigrants’ health depends on their socioeconomic and education levels. Based on these levels, immigrants’ acculturative process will vary and, therefore, their daily habits, access to health resources and even the ability to report health-related problems [29]. It is thus expected that immigrants who had a relatively low exposure to cardiovascular-risk factors in their countries of origin will take on high-risk behaviours from native population in host countries. Nevertheless, results from same levels of exposure to risk factors may vary in individuals or populations from different ethnical origins [30]. Several studies have identified differences in the prevalence of certain diseases related to cardiovascular risks and their subsequent complications in different ethnical groups. For instance, African immigrants suffer more from hypertension and even have more risk of infarction, compared to European groups [31]. Age, gender, geographical origin or place of birth or residence are also social factors that strongly explain the distribution of health and diseases in immigrants. Age is often linked to the suffering of some diseases, that is, certain diseases are highly linked to individuals’ age. There are several studies that conclude that elderly people suffer hypertension, dyslipidemia, diabetes and other diseases related to cardiovascular diseases to a greater extent [32]. Gender has also been confirmed as a variable that affects the development of cardiovascular diseases. Immigrant women are found to have higher risk of developing abdominal obesity and low levels of high-density lipoproteins (HDL) cholesterol, whereas men tend to show higher risks of high blood pressure [33]. However, there are few studies that analyse differences between women and men more specifically in relation to discrimination, psychological distress and cardiovascular risk. For this reason, it is important and innovative to analyse this relation in order to respond to it.

### 1.4. Present Study

The present study aims at determining the impact of discrimination and psychological distress on immigrants’ cardiovascular health. Likewise, potential differences among immigrants based on gender and geographical origin will also be analysed. The effect of age and length of residence in the host country on immigrants’ cardiovascular risk will also be studied. This is an innovative research because it is not common to analyse discrimination and psychological distress variables jointly with the purpose of determining cardiovascular risk in immigrants. Furthermore, sociodemographic variables that affect hypertension and dyslipidemia and, subsequently, the risk of suffering cardiovascular diseases will also be analysed.

**Figure 1:**
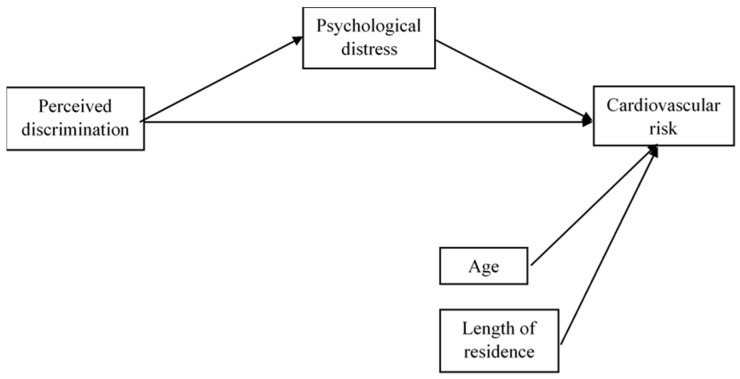
Path model.

Figure 1 shows the network of relations suggested according to the aim of the study. Such relations are specified through the following hypotheses:

**Hypothesis** **1.** *Perceived discrimination predicts the risk of suffering arterial hypertension and dyslipidemia and psychological distress in immigrants; the higher the discrimination, the higher the risk of experiencing psychological distress and developing cardiovascular diseases.*

**Hypothesis** **2.** *Psychological distress mediates the relation between perceived discrimination and cardiovascular risks. The higher the psychological distress, the higher the cardiovascular risk in those immigrants who feel discriminated.*

**Hypothesis** **3.** *Immigrants’ age and length of residence in the host country are co-variables that affect cardiovascular risks.*

**Hypothesis** **4.** *The relation between perceived discrimination, psychological distress and cardiovascular risks will be moderated by gender and ethnical differences (groups from Africa, Eastern Europe and Africa).*

## 2. Materials and Methods

### 2.1. Participants

The sample included 1714 immigrants from Africa (33.2%), Eastern Europe (31.6%) and Latin America (35.2%). The distribution of immigrants in the city represents data from the 2019 census. Regarding gender, 48.7% were men and 51.3% were women. The age range was 15 to 74 years (Mean, M = 33.88; Standard Deviation, SD = 12.31). Data do not differ in any great respect based on immigrants’ geographical origin: the age range for African immigrants is 15–72 years (M = 33.43, SD = 11.26); 17–74 (M = 34.35, SD = 12.46) for Eastern European immigrants; and 15–74 (M = 33.88, SD =13.12) for Latin American immigrants. Data are also similar in both men (range = 16.74, M = 33.49, SD = 12.38) and women (range = 15.72, M = 34.29, SD = 12.23). The average length of time participants had been living in Malaga (Spain) was 10.35 years (SD = 7.30); M = 10.22 (SD = 7.16) and M = 10.50 (SD = 7.42) for men and women, respectively; M = 10.48 (SD = 7.25) in African immigrants, M = 9.80 (SD = 6.77) in Eastern European and M = 10.73 (SD = 7.78) in Latin American immigrants.

### 2.2. Procedure

Data were collected using a random-route sampling and survey methodology. Interviews were conveyed by professionally trained interviewers in all districts of the city. Data were specifically collected in associations, public meeting places, social services centres, locutories, etc. The questionnaires applied to the non-Spanish-speaking immigrants were translated into their mother tongue by native speakers who had a full command of Spanish. All immigrants participated voluntarily, they signed an informed consent form and did not receive any economic retribution for their participation. The study was conducted in accordance with the Declaration of Helsinki, and the protocol was approved by the Ethics Committee of University of Málaga (Spain) (Project identification code CEUMA 37-2016-H).

### 2.3. Measures

Demographic form. Demographic data gathered included age, gender, geographical origin and length of residence in the host country.

Perceived discrimination. The discrimination questionnaire is based on The Experiences of Discrimination Scale designed by Krieger et al. (2005) [34]. This scale is a valid and reliable measure of self-reported discrimination that has been used across many ethnic groups. Participants are asked: ”Over the past year, have you felt discriminated in any of the following contexts?” There are nine situations stated regarding the contexts of education, health, work environment, access to public services, access to housing, police, shops, etc. Responses were recorded using the Likert scale: never (1), sometimes (2), often (3) and many times (4).

Psychological distress. The Spanish version of the Goldberg General Health Questionnaire (GHQ-12) was used [35]. This instrument is used to assess mental health, and it identifies non-psychotic mental health problems. It is an efficient instrument to assess psychological distress in clinical patients and the general population. It consists of 12 items which are answered through a 4-point Likert-type scale ranging from (0) = not at all, to (3) = much more than usual, (e.g., “Have you felt unhappy or depressed?”).

Illness: *Spanish Statistical Office* (2019). This questionnaire comprises a list of 28 illnesses or health problems (hypertension, diabetes, headaches, allergies, etc.) [36]. The present study only included items that were related to hypertension and dyslipidemia. Participants are asked two questions in order to know whether they have experienced some of these two problems over the last 12 months (“Have you had this problem over the last 12 months?” 1. Yes, 2. No).

### 2.4. Data Analysis

**Figure 2:**
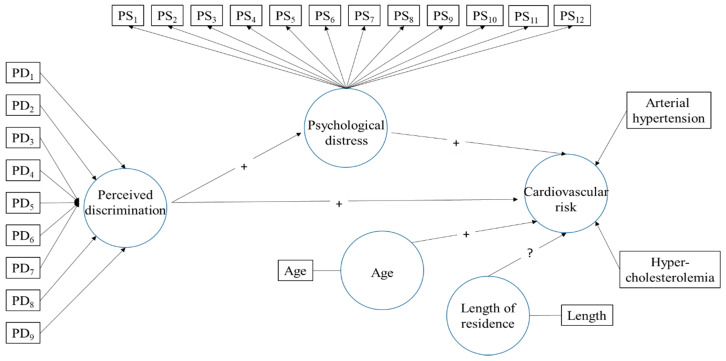
Path diagram of measurement model and structural model. PD_1_-PD_9_ are the nine items of perceived discrimination questionnaire and PS_1_-PS_12_ are the twelve items of psychological distress questionnaire (See Table 1 and Table 2).

Figure 2 shows the path model proposed, which is analysed using the SmartPLS (v.3.3.2) software [37]. This software is particularly suitable for research whose key aim is to predict constructs. It presents less restrictive requirements for the measurement of scales, size of the sample and distribution of data. As it can be observed in the model, the independent variable perceived discrimination and the dependent variable cardiovascular risk are considered formative constructs. The mediating variable of psychological distress is defined as a reflective construct. As we know, formative measurements are latent constructs formed by measurement indicators, where these are the cause or precedent of the construct. On the contrary, in reflective measurements, indicators of the latent variable compete and represent a manifestation of the latent variable [38]. In contrast with the former, the construct causes the measurement of the indicative variables. This difference can be seen in Figure 1, represented by the different directionality of the arrows connecting constructs to their indicators. In the formative model, each of the nine indicators of discrimination and the two indicators of cardiovascular risk represent a dimension of the meaning of the latent variable in question—removing one indicator makes the variable lose part of its meaning. For this reason, it is highly important for indicators to cause the construct. In this case, outer weights are estimated through a multiple regression in which perceived discrimination or cardiovascular risk represent a dependent variable and their linked indicators are independent variables. Psychological distress has 12 indicators, and each one of its outer loadings are estimated through a simple regression on each item based on the construct of psychological distress.

Figure 2 also shows the relations of the structural model, in which perceived discrimination has direct relations with psychological distress and cardiovascular risk, as well as an indirect relation with the later through stress, which acts as a mediating variable. Finally, there is also a direct relation between stress and risk. Apart from these relations, the model also includes two co-variables: age and length of residence in Malaga. These co-variables are variables of statistical control of the theoretical relations proposed.

The assessment of the measurement model (formative and reflective) and the structural model were carried out through the PLS-SEM algorithm of the SmartPLS software. The process of full bootstrapping (5000 samples), Bias-Corrected and Accelerated Bootstrap, the two-tailed test and the level of significance of 5% were used.

The model is also tested based on the two moderating variables of geographic origin of immigrants (African, Latin American and Eastern European) and gender (men and women). To this end, the same model for both sub-groups was calculated and a subsequent Multigroup Analysis (MGA) to know whether there were significant differences between them was also conveyed. The PLS-MGA approach was applied to geographic origin and gender. The three groups of origin and the two groups of gender will be assessed based on the significant differences in the estimation of parameters (for instance, weights, loadings and path coefficients). Each group of origin was thus compared to the other two as well as men were compared to women.

## 3. Results

Results from the measurement model are presented, followed by the results from the structural model. The measurement model is exclusively presented for the total sample, in order to avoid redundancy, while the structural model shows the results of the total group of immigrants and the results, separately, based on geographic origin and gender.

Table 1 shows the results from the reflective measurement model of psychological distress. As it can be seen, loadings from all items are statistically significant, although their magnitudes vary from the maxim of 0.743 of the indicator (“Do you feel reasonably happy considering all circumstances?”) to the minimum of 0.373 of the item (“Have you been able to concentrate well?”). Reliability indicators for each indicator are also appropriate. Regarding global rates of reliability and validity of the construct, the latent variable of psychological distress has good composite reliability values, Cronbach’s Alpha and Rho A, thus being significant in all cases. Regarding the discriminant validity, the Heterotrait–Monotrait ratio (HTMT) for the relation between age and stress is 0.046, CI, Confidence Interval, 95% [0.038, 0.094]. For the relation between length of residence and stress, the value is 0.132, CI 95% [0.087, 0.182]. For both cases, confidence intervals of HTMT do not include value 1, which would indicate poor validity. Overall, it is worth noting that all reliability and validity criteria for the assessment of the measurement model of psychological distress are met.

Table 2 shows the results from the formative measurement model. Only three of the weights of perceived discrimination indicators are statistically significant. However, loadings from some of the non-significant indicators are high; all of them are above 0.50, except for items 7 and 8. When a high number of formative indicators are used for a construct, it is common for some of them to not be significant. This does not imply poor quality of the measurement model, and the total contribution made by each indicator to its construct must be taken into consideration. Removing non-significant indicators would change the whole significance of the latent variable. The two formative indicators of cardiovascular risk (arterial hypertension and hypercholesterolemia) have significant weights, and their total contributions to the construct are high.

Table 3 shows the results from the structural model for the total sample of immigrants and for the samples of participants from Africa, Latin America and Eastern Europe, as well as results based on gender.

According to the total sample, perceived discrimination is a good predictor of psychological distress as well as of cardiovascular risk, although to a lesser extent. There is a statistically significant positive relation between perceived discrimination and psychological distress. The relation with cardiovascular risk is also statistically significant, but it is lower. Psychological distress also predicts cardiovascular risk moderately. Higher age is related to higher cardiovascular risk, whereas the length of residence has no significant relation. The mediating effect of psychological distress is positive and statistically significant; however, it is also low. Overall, higher discrimination relates to higher cardiovascular risk. This relation is increased when psychological distress increases. The R^2^ for psychological distress has a value of 0.128 (*t* = 6.72, *p* < 0.001, CI 95% [0.101, 0.174]) and cardiovascular risk has a R^2^ = 0.124 (*t* = 7.72, *p* < 0.001, CI 95% [0.097, 0160].

Regarding the comparison between the three geographic origins, the effect of discrimination on cardiovascular risk is notably higher in the case of Latin American immigrants. The magnitude of their coefficient is almost double as compared to the other two groups. In this group, the coefficient that relates length of residence and cardiovascular risk is also positive and statistically significant. For the other two groups, this relation is negative and non-significant. There are no differences between the three groups in the coefficient that relates discrimination and psychological distress and the same applies to the mediating effect of psychological distress. The PLS Multigroup analysis indicated that none of the differences found between coefficients from the three groups were statistically significant.

Discrimination was observed to better predict cardiovascular risk in women than in men. The relation of these coefficients in the case of men was very low and non-significant. However, discrimination affects psychological distress in men slightly more than in women. Age affects both genders in the same way. Nevertheless, this is not the case for the variable of length of residence in the host country, which is different for men and women, although there is no statistical significance in any group. The mediating effect of psychological distress is very similar in both groups. The PLS-MGA analysis reveals that the only difference that is on the limit of statistical significance is the coefficient that relates discrimination and cardiovascular risk. This difference in coefficients has a value of 0.157 (*p* = 0.056).

## 4. Discussion

The present study aims at analysing the relation between perceived discrimination, psychological distress and cardiovascular risk in immigrants. The measurement model is appropriate and, overall, all reliability and validity criteria of the reflective measurement model related to psychological distress are met. The same applies to the formative measurement model of perceived discrimination. Likewise, formative indicators of cardiovascular risk, arterial hypertension and hypercholesterolemia have significant weights, and their absolute contributions to the construct are high. The results from the structural model are presented below. This model was used to analyse the relation between perceived discrimination, psychological distress and cardiovascular risk. In this model, age and length of residence are considered co-variables, and ethnical differences and gender are considered moderating variables. In this case, the model works quite well for the general sample of immigrants, and results are moderated to a certain extent by gender and ethnical origin.

Results obtained from this research denote that discrimination experienced by immigrants predicts their psychological distress and cardiovascular risk. Those immigrants who have experienced more discrimination in the host country seem to suffer higher levels of stress due to the acculturative process. Along with all the challenges they face in their new cohabitation contexts, they also feel not integrated in the community, which affects their health and well-being [4,39]. These results confirm the first hypothesis of the study and match the findings from other research. Such studies reveal that immigrants who express feeling discriminated in the host country are more likely to suffer diseases such as hypertension, hypercholesterolemia, diabetes and obesity, among others [5,7,8]. In order to counteract the negative effect on health caused by discrimination as well as to reduce the cardiovascular risk related to it, it would be necessary to design social inclusion policies and interventive strategies that would promote the social inclusion of this collective [9].

Psychological distress predicts cardiovascular risk moderately. It also mediates between discrimination and arterial hypertension and hypercholesterolemia, thus confirming the second hypothesis of the study. In general terms, higher discrimination relates to higher cardiovascular risk. This relation is increased when psychological distress increases. Therefore, migrating might have a negative impact on immigrants’ health due to multiple physical and psychosocial tensions they suffer throughout the migration process, which leads them to suffer from acculturative stress [23]. Some of the multiple stressors immigrants must face include adapting to a new culture and its social norms, overcoming the language barrier, precarious working conditions, economic difficulties, obstacles to legalise their situation in the host country and the loss of social networks of support [20,40,41,42,43]. These factors can lead to suffer discrimination, high levels of stress and increase risk behaviours in immigrants, which finally affect their health [44,45,46].

Cumulative exposure to chronic stress can lead to a decline in the health of those immigrants who have lived longer in the host country [47]. As they settle in the host country and adopt new behaviours such as smoking, drinking alcohol or eating fast food, their risks of developing cardiovascular diseases, diabetes and obesity increase [24,25,26,27,28]. The present study has not found results in the general sample to confirm that the higher the length of residence in the host country, the higher the cardiovascular risk. However, this relation has been found in the group of Latin American immigrants, where the coefficient relating length of residence and risk of developing arterial hypertension and hypercholesterolemia is positive. On the contrary, Eastern European and African immigrants showed opposite results, although non-significant, in the sense of longer length of residence relating to lower cardiovascular risk. In any event, the differences in the magnitude of coefficients from the three groups are not statistically significant. According to the results from the present study and previous literature, it can be concluded that there is no absolute consent on how the length of residence in host countries affects the health of immigrants. Some studies suggest that as immigrants settle in the host country, their health would improve [48]. However, other studies suggest otherwise and highlight the decline in the general health of immigrants and their risk of developing cardiovascular diseases [49,50].

A significant relation between age and cardiovascular risk was found, in such a way that older immigrants would be more likely to suffer hypertension and hypercholesterolemia and, ultimately, higher risk of cardiac failure, cerebrovascular accident or myocardial infarction. This finding matches previous studies [32] as well as it confirms what occurs with native populations, where the increase of age is related to an increase in health problems in general [51]. These findings confirm the third hypothesis of the study, in which the relation between discrimination perceived, psychological distress and cardiovascular risk varies based on age.

Regarding the conclusions drawn from the variable of geographic origin, the effect of discrimination on cardiovascular risk is considerably higher in the case of Latin American immigrants, whose weight almost doubles the results from the other two groups of immigrants (Eastern European and African). Even though the differences found are not statistically significant, they are worth noting. These findings match the results from other studies in which the difference in geographic origin can determine the risk of suffering certain diseases [30,31]. For instance, some studies have identified that African immigrants are more likely to suffer hypertension and have more risk of infarct, as opposed to European groups. However, Latin American immigrants were not included in these studies [31]. It would be interesting, therefore, to further analyse the results obtained from the present study regarding the sample of Latin American immigrants and the higher effect of discrimination on cardiovascular risk. No differences were found between the three ethnic groups regarding the remaining variables of the study (relation between perceived discrimination and psychological distress and the mediating effect of the latter). The aim is to take a closer look at these relations, given the fact that immigrants face stress with different behavioural and cognitive responses. This can be key to know whether stress affects their health to a greater or lesser extent [52].

Regarding the differences found in terms of gender, discrimination is observed to better predict cardiovascular risk in women as opposed to men. However, discrimination affects psychological distress in men to a greater extent. This difference in women was observed to not be statistically significant. The fourth hypothesis of the study is thus confirmed, and it matches results from previous studies, which suggest that there is a specific relation between perceived discrimination and cardiovascular risk based on gender [53]. For instance, the Jackson Heart Study [54] confirms a significant relation between discrimination and hypertension in women from different geographic origins, as opposed to men, where this did not occur. Women with high exposure to discrimination were more likely to develop hypertension, while the probability of developing hypertension in those women with low or non-existent perception of discrimination decreased considerably. Furthermore, this study noted that discrimination also affects what is known as “burden of discrimination” in a distinctive way based on gender. While no significant relations were found in women, the burden of discrimination was tightly linked to hypertension in men [54]. It seems, therefore, that the fact of being a man or a woman moderates both the direct relation between perceived discrimination and cardiovascular risk and the indirect relation between perceived discrimination, psychological distress and cardiovascular risk. Major differences based on gender were observed, with a notable effect of psychological distress in men. Incidentally, age affects both genders in the same way, meaning that the higher the age, the higher the cardiovascular risk for both men and women.

### 4.1. Future Lines of Research

As it has been proved throughout the present study, discrimination is a risk factor for the health of immigrants, which has been broadly studied in literature [55]. However, studies on the protective effects of cardiovascular health in immigrants are scarce, which is why the subject should be further explored in future research. Some authors suggest that disease prevention is affected by access to information and social networks. According to this notion, social support could mediate the experiences of discrimination and promote the search for health care in the community, as well as self-care actions [56,57]. As noted by Kokab et al. (2008), the decision of implementing preventive behaviours of intervention in health care is tightly related to family and community support, particularly in the case of non-communicable diseases such as cardiovascular diseases [58]. Therefore, intervention programmes in health care should take into consideration such social and community dynamics.

Multiple studies suggest that negative experiences suffered by immigrants in host countries significantly affect their health, with notable differences based on family support and community cohesion [47]. Shared experiences of immigration, social support and inclusion and socio-economic factors play a key role in understanding and self-managing diabetes in immigrants [59], managing arterial hypertension [60] and the risk of suffering cardiovascular diseases [61]. Social relations have a favourable effect in immigrants’ health, in such a way that family relations, support from friends and relatives and the cohesion within the neighbourhood are indicators of optimal levels of health [62]. Research conducted with immigrants who live in Spain confirms a better health in those immigrants with strong support networks and a high satisfaction with the support received [63]. By the same token, the model suggested by Fernández, Silván-Ferrero, Molero, Gaviria and García-Ael (2015) includes social support as a mediating element between perceived discrimination by immigrants and their personal well-being [64]. Therefore, it is important to analyse in future research the effect of social support on immigrants, acting as a protective factor against the risk of developing certain cardiovascular diseases as well as its role as a potential mediating variable between discrimination, psychological distress and cardiovascular risk.

The line of research might also be broadened to include more specific analyses on the effect of the socio-economic level on the cardiovascular health of immigrants. Some studies conducted on immigrants with good socio-economic status show that these immigrants present a better cognitive and physical condition, while those immigrants with lower socio-economic and education levels show poorer health [65].

It would be of great interest to analyse the specific effect of psychological distress in children and young immigrants (16–20 years). The psychological responses through which children and teenagers go during the migration process are multiple and varied, since the situations they experience are emotionally highly shocking. Research shows that very often, the migration process is a traumatic experience for young immigrants, which affects their interpersonal relations and it is linked to high levels of post-traumatic stress [66,67]. Some of the most relevant problems that children and young immigrants face are separation anxiety, depressive disorders and post-traumatic stress [68]. Special attention should be paid to children and young immigrants during such a vital stage to help them face the psychological problems caused by the migration process.

Finally, it would also be of interest to delve into the variables analysed and, through a qualitative study, confirm whether the negative effects of discrimination and psychological distress decrease based on ethnical differences between the different groups of immigrants and the native population as well as according to other sociodemographic variables such as gender. To study this comparison between native population and immigrants may provide interesting results on the negative effects of perceived discrimination and psychological distress. Although some literature gathers the “Immigrants Paradox” [69], according to which immigrants are healthier than local populations, several studies nuance such paradox: immigrants might have better or similar physical health than native populations, but they show worse mental health, which might be caused by the stress suffered during the migration process and the discrimination suffered in the host country [70,71].

### 4.2. Limitations of the Study

The research of the present study was conducted using quantitative methodology, and it seems necessary to combine other study methods that are more qualitative in order to complete the results obtained, such as comprehensive interviews with immigrants or their life stories. Likewise, given the correlational methodology used, no causality relations can be established between variables. By conducting a longitudinal analysis, some light might be shed on how health problems evolve and which are the risk factors in immigrants. Furthermore, the present study was conducted in Spain, which has large numbers of immigrants, and the sample is rather large, but it would be of interest to carry out the same study in other countries and with immigrants from different origins in order to confirm whether similar results are achieved.

## 5. Conclusions

In summary, the present study is innovative because it analyses the relation between discrimination, psychological distress and cardiovascular risk in immigrants from different ethnic origins who live in Spain. Differences based on gender, age and length of residence have also been studied. Results show that discrimination acts as a predictor of arterial hypertension and hypercholesterolemia, as well as psychological distress, which also mediates between discrimination experienced by immigrants and their risk of developing certain cardiovascular diseases. Regarding the comparison between the three geographical origins, there are no conclusive results. However, data suggest that the effect of discrimination on cardiovascular risk is higher in Latin American immigrants than in the other two groups. The coefficient that relates length of residence and cardiovascular risk is also higher for this group. Significant differences regarding the effect of age on the variables studied were also found. It is important to highlight the differences found based on gender, since discrimination predicts cardiovascular risk to a higher extent in women than in men. These findings can be considered innovative because variables that are usually studied separately have been jointly analysed, and a large sample of immigrants from different geographic origins has been used. These findings are also relevant for the design of preventive programmes with immigrants, since they can be helpful towards knowing which intervention strategies are more efficient to prevent cardiovascular risk in immigrants.

## Figures and Tables

**Table 1:** Results summary from the reflective measurement model of psychological distress.

Latent Variable	Indicators	Loadings	*t* Value	*p* Value	95% Confidence Interval		Indicator Reliability	Items
					Lower	Upper		
Psychological Distress	PS_1_	0.373	11.631	0.000	0.309	0.434	0.139	Have you been able to concentrate well?
	PS_2_	0.690	35.421	0.000	0.649	0.726	0.476	Have your concerns made you lose much sleep?
	PS_3_	0.619	26.545	0.000	0.569	0.662	0.383	Have you felt you are leading a useless life?
	PS_4_	0.628	27.596	0.000	0.581	0.670	0.394	Have you felt unable to make decisions?
	PS_5_	0.578	26.044	0.000	0.532	0.619	0.334	Have you felt constantly stressed and under tension?
	PS_6_	0.586	24.595	0.000	0.536	0.630	0.343	Have you had the feeling of not being able to overcome difficulties?
	PS_7_	0.463	16.082	0.000	0.406	0.517	0.214	Have you been able to enjoy everyday activities?
	PS_8_	0.647	33.321	0.000	0.606	0.682	0.418	Have you been able to face your problems appropriately?
	PS_9_	0.689	40.245	0.000	0.653	0.721	0.475	Have you felt unhappy or depressed?
	PS_10_	0.584	25.032	0.000	0.536	0.628	0.341	Have you lost confidence in yourself?
	PS_11_	0.571	23.887	0.000	0.521	0.615	0.326	Have you felt like a useless person?
	PS_12_	0.743	49.599	0.000	0.711	0.770	0.552	Do you feel reasonably happy considering all circumstances?
AVE = 0.370			34.953	0.000	0.349	0.391		
Composite Reliability = 0.873			164.924	0.000	0.862	0.883		
Rho A = 0.858			128.750	0.000	0.845	0.871		
Cronbach’s Alpha = 0.844			121.381	0.000	0.830	0.857		

AVE, average variance extracted; Rho A, index of composite validity.

**Table 2:** Formative constructs outer weights significance testing results of perceived discrimination and cardiovascular risk.

Formative Constructs	Formative Indicators	Outer Weights	Outer Loadings	*t* Value	*p* Value	95% Confidence Interval		Items
						Lower	Upper	
Perceived Discrimination	PD_1_	0.340	0.771	3.625	0.000	0.149	0.510	Work environment (access to work, salary, promotion, training, dismissal, balancing work and family life)
	PD_2_	0.410	0.812	4.273	0.000	0.212	0.585	Access to public services (education, health care, subsidies, means of transport)
	PD_3_	0.085	0.649	0.915	0.360	−0.099	0.271	Attention and treatment from the Public Administration (citizen’s assistance offices, information, civil servants)
	PD_4_	0.049	0.536	0.548	0.584	−0.131	0.223	Treatment by the police
	PD_5_	0.138	0.676	1.452	0.147	−0.052	0.320	Access to housing
	PD_6_	0.250	0.672	2.676	0.007	0.060	0.425	At shops, bars and other private services
	PD_7_	−0.062	0.243	0.524	0.600	−0.294	0.174	In your family (from your partner)
	PD_8_	0.040	0.292	0.360	0.719	−0.177	0.248	In your family (from someone who is not your partner)
	PD_9_	0.048	0.645	0.501	0.617	−0.141	0.237	In the street, treatment from people
Cardiovascular Risk	Hypertension	0.686	0.822	11.600	0.000	0.564	0.794	Arterial hypertension
	Cholesterol	0.586	0.745	8.956	0.000	0.452	0.707	Hypercholesterolemia

**Table 3:** Path model coefficients for the total group of immigrants, based on geographic origin and gender.

Effects	Path Coefficients	*t* Value	*p* Value	95% Confidence Intervals		Significance (*p* < 0.05)? **
				Lower	Upper	
Total						
*Direct effect*						
Perceived Discrimination → Psychological Distresss	0.363	13.701	0.000	0.320	0.423 *	Yes
Perceived Discrimination → Cardiovascular Risk	0.134	4.272	0.000	0.074	0.198 *	Yes
Psychological Distresss → Cardiovascular Risk	0.118	4.353	0.000	0.063	0.171 *	Yes
Age → Cardiovascular Risk	0.275	10.607	0.000	0.223	0.324 *	Yes
Length of Residence → Cardiovascular Risk	0.020	0.611	0.541	−0.044	0.084	No
*Indirect effect*						
Perceived Discrimination → Psychological Distresss → Cardiovascular Risk	0.043	4.027	0.000	0.023	0.065 *	Yes
Africa						
*Direct effect*						
Perceived Discrimination → Psychological Distresss	0.385	9.616	0.000	0.334	0.491 *	Yes
Perceived Discrimination → Cardiovascular Risk	0.127	2.221	0.026	0.024	0.248 *	Yes
Psychological Distresss → Cardiovascular Risk	0.107	2.141	0.032	0.004	0.200 *	Yes
Age → Cardiovascular Risk	0.293	6.328	0.000	0.197	0.380 *	Yes
Length of Residence → Cardiovascular Risk	−0.030	0.505	0.614	−0.141	0.086	No
*Indirect effect*						
Perceived Discrimination → Psychological Distresss → Cardiovascular Risk	0.041	1.973	0.048	0.002	0.085 *	Yes
Latin America						
*Direct effect*						
Perceived Discrimination → Psychological Distresss	0.377	8.707	0.000	0.310	0.479 *	Yes
Perceived Discrimination → Cardiovascular Risk	0.213	3.453	0.001	0.093	0.333 *	Yes
Psychological Distresss → Cardiovascular Risk	0.116	2.569	0.010	0.025	0.198 *	Yes
Age → Cardiovascular Risk	0.290	6.808	0.000	0.206	0.370 *	Yes
Length of Residence → Cardiovascular Risk	0.148	2.869	0.004	0.041	0.245 *	Yes
*Indirect effect*						
Perceived Discrimination → Psychological Distresss → Cardiovascular Risk	0.044	2.283	0.022	0.010	0.085 *	Yes
Europe						
*Direct effect*						
Perceived Discrimination → Psychological Distresss	0.395	7.274	0.000	0.301	0.516 *	Yes
Perceived Discrimination → Cardiovascular Risk	0.112	1.269	0.204	−0.054	0.286	No
Psychological Distresss → Cardiovascular Risk	0.109	2.022	0.043	0.000	0.210	No
Age → Cardiovascular Risk	0.254	4.997	0.000	0.149	0.348 *	Yes
Length of Residence → Cardiovascular Risk	−0.052	1.072	0.284	−0.145	0.048	No
*Indirect effect*						
Perceived Discrimination → Psychological Distresss → Cardiovascular Risk	0.043	1.894	0.058	0.000	0.090	No
Women						
*Direct effect*						
Perceived Discrimination → Psychological Distresss	0.341	8.346	0.000	0.272	0.432 *	Yes
Perceived Discrimination → Cardiovascular Risk	0.186	3.413	0.001	0.085	0.303 *	Yes
Psychological Distresss → Cardiovascular Risk	0.116	3.300	0.001	0.044	0.184 *	Yes
Age → Cardiovascular Risk	0.258	7.052	0.000	0.185	0.328 *	Yes
Length of Residence → Cardiovascular Risk	−0.029	0.621	0.535	−0.117	0.067	No
*Indirect effect*						
Perceived Discrimination → Psychological Distresss → Cardiovascular Risk	0.040	2.952	0.003	0.015	0.068 *	Yes
Men						
*Direct effect*						
Perceived Discrimination → Psychological Distresss	0.404	11.498	0.000	0.354	0.491 *	Yes
Perceived Discrimination → Cardiovascular Risk	0.067	1.439	0.150	−0.023	0.163	No
Psychological Distresss → Cardiovascular Risk	0.143	3.218	0.001	0.052	0.226 *	Yes
Age → Cardiovascular Risk	0.293	8.051	0.000	0.220	0.363 *	Yes
Length of Residence → Cardiovascular Risk	0.066	1.420	0.156	−0.023	0.159	No
*Indirect effect*						
Perceived Discrimination → Psychological Distresss → Cardiovascular Risk	0.058	2.969	0.003	0.022	0.098 *	Yes

* 95% CI, confidence interval, does not include 0; ** We refer to the bootstrap confidence intervals for significance testing.
